# Temporal Dynamics of the Default Mode Network Characterize Meditation-Induced Alterations in Consciousness

**DOI:** 10.3389/fnhum.2016.00372

**Published:** 2016-07-22

**Authors:** Rajanikant Panda, Rose D. Bharath, Neeraj Upadhyay, Sandhya Mangalore, Srivas Chennu, Shobini L. Rao

**Affiliations:** ^1^Cognitive Neuroscience Center, National Institute for Mental Health and NeurosciencesBangalore, India; ^2^Department of Neuroimaging and Interventional Radiology, National Institute for Mental Health and NeurosciencesBangalore, India; ^3^Department of Neurology and Psychiatry, Sapienza University of RomeRome, Italy; ^4^School of Computing, University of KentChatham Maritime, UK; ^5^Department of Clinical Neurosciences, University of CambridgeCambridge, UK

**Keywords:** default mode network, microstate, DMN-microstate, simultaneous EEG-fMRI, meditation

## Abstract

Current research suggests that human consciousness is associated with complex, synchronous interactions between multiple cortical networks. In particular, the default mode network (DMN) of the resting brain is thought to be altered by changes in consciousness, including the meditative state. However, it remains unclear how meditation alters the fast and ever-changing dynamics of brain activity within this network. Here we addressed this question using simultaneous electroencephalography (EEG) and functional magnetic resonance imaging (fMRI) to compare the spatial extents and temporal dynamics of the DMN during rest and meditation. Using fMRI, we identified key reductions in the posterior cingulate hub of the DMN, along with increases in right frontal and left temporal areas, in experienced meditators during rest and during meditation, in comparison to healthy controls (HCs). We employed the simultaneously recorded EEG data to identify the topographical microstate corresponding to activation of the DMN. Analysis of the temporal dynamics of this microstate revealed that the average duration and frequency of occurrence of DMN microstate was higher in meditators compared to HCs. Both these temporal parameters increased during meditation, reflecting the state effect of meditation. In particular, we found that the alteration in the duration of the DMN microstate when meditators entered the meditative state correlated negatively with their years of meditation experience. This reflected a trait effect of meditation, highlighting its role in producing durable changes in temporal dynamics of the DMN. Taken together, these findings shed new light on short and long-term consequences of meditation practice on this key brain network.

## Introduction

The grand challenge of characterizing the dynamic neural substrate underlying human consciousness has captured the interest of many researchers cutting across disciplinary boundaries and covering altered states ranging from sleep, meditation, hypnosis, anesthesia, coma, disorders of consciousness, delirium tremens, psychoses, etc. Normal consciousness is thought to require both wakefulness and arousal, and several neuro scientifc studies conceptualize wakefulness as a continuum with different levels of awareness (Grill-Spektor et al., 2000; Bar et al., 2001; Kouider et al., 2010). Resting state functional magnetic resonance imaging (rsfMRI) has been used to study the neural correlates of conscious awareness in normal and altered states of consciousness. In particular, this research enterprise has highlighted and progressively refined our understanding of the so-called default mode network (DMN) of the brain, consisting of precuneus/posterior cingulate cortex (PCC), medial frontal cortex (mPFC), the temporoparietal junction (TPJ) and hipocampal formation including parahipocampal cortex, as a key neural correlate of consciousness (Buckner et al., 2008). In particular, researchers have studied changes in the DMN as a function of meditative and introspective cognitive states like day dreaming, mind wandering, and autobiographical memory retrieval (Baerentsen et al., 2010; Baron Short et al., 2010; Hasenkamp et al., 2012; Garrison et al., 2013). These investigations into the neurophenomenology of meditation have found PCC deactivation to be associated with “undistracted awareness” and “effortless doing”, with PCC activation being linked to “distracted awareness” and “controlling” in experienced meditators (Garrison et al., 2013). This evidence is in line with the contrasting approaches in two major types of meditation, which either emphasize focused attention or open monitoring. Focused attention meditation aims to reduce mind wandering by concentrating on tasks like breath, sounds or mental imagery, while open monitoring meditation practice encourages mind wandering, and makes one aware of this process (Xu et al., 2014). Extensive study of the DMN in health and disease have also found correlations between DMN connectivity and sleep (Fukunaga et al., 2006; Horovitz et al., 2008; Picchioni et al., 2008), anesthesia (Vincent et al., 2007), disorders of consciousness (Fernández-Espejo et al., 2012; Guldenmund et al., 2012).

While rsfMRI has enabled us to have a fine-grained spatial understanding of the DMN in these states of consciousness, conventional analytical approaches are not best suited to measure the temporal dynamics of its activity, especially during meditation-induced alterations in the state of consciousness. To better understand the temporal dynamics of the DMN, researchers have developed a range of techniques, including time resolved resting fMRI analysis where DMN connectivity is assessed multiple times using sliding temporal windows (Chang and Glover, 2010; Hutchison et al., 2013; Leonardi et al., 2013; Allen et al., 2014; Zalesky et al., 2014). Alternative techniques to obtain temporal detail with fMRI include identification of temporal functional modes using temporal independent component analysis (ICA; Smith et al., 2012), modified seed to voxel based connectivity to define coactivation patterns (Liu and Duyn, 2013) and the recent innovation driven coactivation pattern (Karahanoglu and Van De Ville, 2015). While these methods have attempted to capture the temporal dynamics of the DMN, the time scales of the observed fluctuations in its activity vary from tens of seconds to few minutes in fMRI based studies (Chang and Glover, 2010; Handwerker et al., 2012; Hutchison et al., 2013). In contrast, modeling of electromagnetic brain dynamics using electroencephalography (EEG) and MEG suggest that these neuronal fluctuations called microstates have durations of 100–200 ms (Brandeis and Lehmann, 1989; Pascual-Marqui et al., 1995; Michel et al., 2004; Lehmann et al., 2010; Baker et al., 2014). The concept of microstates was first proposed and demonstrated by Lehmann et al. (1987) when they described brain states that remain stable for 80–120 ms before rapidly evolving into another quasi-stable microstate. The most common parameters used to quantify microstate dynamics are duration or lifespan, which is the average length of time each microstate remains stable whenever it appears. Another useful parameter is frequency of occurrence, which is the average number of times per second that the microstate becomes dominant (Lehmann et al., 1987). Most microstate studies reports four classic microstates which can explain more than 70% of the variation in the scalp topographies manifesting in EEG time series (Tei et al., 2009; Khanna et al., 2015), which have been found to be correlated with rsfMRI networks associated with phonological processing, visual processing, the salience network, and attentional switching (Mantini et al., 2007; Britz et al., 2010). Other studies have reported a higher number of microstates (10–13) with different analysis methods (Musso et al., 2010; Yuan et al., 2012), some of which correlate with the DMN. Here, we refer to the “DMN microstate” as the EEG microstate which correlated maximally with the DMN identified with fMRI. Such microstate analyses have been applied to the study of meditation, where increased duration of microstates has been reported in EEG-based studies in Chan-meditators and Ch’anMo’chao, or Vipassana meditators (Faber et al., 2005; Lo and Zhu, 2009; Tei et al., 2009). Microstate parameters have also been shown to be modulated by psychiatric disorders (Dierks et al., 1997; Lehmann et al., 2005; Kikuchi et al., 2011; Nishida et al., 2013) and even by personality type (Schlegel et al., 2012).

In this study, we analyzed the DMN microstate to understand the mechanisms of meditation-induced alterations in consciousness. By contrasting healthy controls (HCs) at rest against expert meditators at rest and during meditation, we explored both state and trait changes in DMN-microstate dynamics produced by meditation with a hypothesis that these could cause differential alterations in its duration and frequency. The state changes felt during meditation are usually described as a deep sense of calm peacefulness, cessation or slowing of mind’s internal dialog and conscious awareness merging completely with the object of meditation (Brown, 1977; Wallace, 1999). Alongside, long-term expertise in meditation also produces durable changes in neural dynamics, with improvements in mental and physical health presumably due to its trait effects (Chiesa and Serretti, 2010, 2011; Hofmann et al., 2010). Here, we describe changes in the spatial configuration of the DMN as a function of meditation, and show that state and trait influences on the temporal dynamics of the DMN microstate can indeed be dissociated.

## Materials and Methods

### Participants

This was a prospective study conducted at a tertiary neurological institute, the National Institute of Mental Health and Neurosciences (NIMHANS) in Bangalore, India. The study was performed after obtaining informed written consent from the participants. They were recruited as healthy participants in the multi-institutional study on cognitive networks. Ethical approval was obtained from the institutional ethics committee for studies involving humans, convened by NIMHANS (No.NIMHANS/69th IES/2010). The meditator cohort included 20 Raja Yoga expert meditators (male; age: 35 ± 7.9 years, years of education: 15.4 ± 1.6 years, right handed) from the Brahma Kumaris Spiritual Organization, all of them with more than 10 years of Raja yoga meditation practice. Raja yoga meditation involves internally visualizing a glowing star as rays emerging between the eye brows and thus could be considered a type of focused attention type of meditation practice. All participants reported that they spent 1.68 ± 0.59 h in meditation per day in the last 10–22 years (15.2 ± 3.54). They also reported having a cumulative experience of 11332.5 ± 6009.86 h of meditation practice (cumulative experience calculated by combining the numbers of hours per day with years of meditation practice) in their life. Twenty HCs were also recruited for the study. The control participants were matched with the meditator cohort by age, gender, education and ethnicity (male; age: 29 ± 6.8 years, years of education: 16.1 ± 1.1 years, right handed) and both the groups were comparable. None of controls had experience in any type of regular meditative practices. Both meditators and control participants were multilingual (languages known 3.38 ± 0.58), with kannada as first language. None of the participants had any history of neurological or psychiatric illnesses, or prior trauma, and were not on any chronic medications that could affect the experiment.

### Experiment Design

The study design was explained to the subjects and instructions were given before performing the EEG-fMRI data collection in the MRI scanner. The participants (both controls and meditators) were instructed to lie awake, with their eyes closed in a relaxed state within the MRI gantry. They were advised to refrain from any cognitive, language or motor tasks during the acquisition. Ear plugs were given to reduce scanner induced noise. Initially a 4.24 min structural MRI was recorded. This allowed them to get familiarized with the MRI environment before starting the rest and meditation session. A single resting EEG-fMRI was obtained in HCs (9.24 min). However in meditators two serial (9.24 min each) resting EEG-fMRI were recorded, one during resting wakefulness, followed immediately by one during meditation. During a *post hoc* interview, all meditators reported that they could satisfactorily achieve the meditative state inside the scanner. This was also subsequently confirmed with analysis of the EEG wave form, where we visually inspected the EEG time series in the time and spectral domains to identify an increase in alpha oscillations during meditation, consistent with previous studies which have reported this increase (Takahashi et al., 2005; Lagopoulos et al., 2009).

### Data Acquisition

#### EEG Data Acquisitions

EEG data were recorded using a 32-channel MR-compatible EEG system (Brain Products GmbH, Gilching, Germany). The EEG cap (BrainCap MR, Brain Products) consisted of 31 scalp electrodes placed according to the international 10–20 system electrode placement and one additional electrode dedicated to the ECG which was placed on the back of the subject. Data were recorded relative to an FCz reference and a ground electrode (GND) according to 10–20 electrode system. Data was sampled at 5000 Hz to enable removal of MRI gradient artifact. The impedance between electrode and scalp was kept below 5 kΩ. EEG was recorded using the Brain Recorder software (Version 1.03, Brain Products). To prevent the movement of the subjects’ head, we placed sufficient padding between the head and the head coil of the MRI scanner. Total time for each rest EEG recoding was same as rsfMRI recording (9.24 min), and time locked to each other.

#### fMRI Data Acquisition

rsfMRI was acquired using a 3T scanner (Skyra, Siemens, Erlangen, Germany). One hundred and eighty-five volumes of gradient-echo, Echo-Planar Images (EPI) were obtained using the following EPI parameters: 36 slices, 4 mm slice thickness in interleaved manner with an field of view (FOV) of 192 × 192 mm, matrix 64 × 64, repetition time (TR) 3000 ms, echo time (TE) 35 ms, re-focusing pulse 90°, matrix- 256 × 256 × 114, voxel size-3 × 3 × 4 mm. We also acquired a three dimensional magnetization prepared rapid acquisition gradient echo (3D MPRAGE) sequence for anatomical information (with the voxel size 1 × 1 × 1 mm, 192 × 192 × 256 matrix) for better registration and overlay of brain activity.

### Data Analysis

#### EEG Artifact Removal and Preprocessing

Raw EEG data was processed offline using BrainVision Analyzer software version 2 (Brain Products GmbH, Gilching, Germany). The gradient artifacts were corrected according to Allen et al. (2000). A moving average width of 20 MR volumes (TRs) was used for gradient correction. Corrected EEG data were filtered using a high-pass filter at 0.03 Hz and a low-pass filter at 75 Hz. The data was then down-sampled to 250 Hz. Ballistocardiogram (BCG) artifacts were removed from the EEG using an averaged subtraction method using heartbeat events (R peaks; Allen et al., 1998, 2000; Goldman et al., 2000), implemented in BrainVision Analyzer 2. Once gradient and BCG artifacts were removed, the data were downsampled to 250 Hz. They were visually inspected for artifacts resulting from muscular sources or head movement artifact and any epoch containing any channel varying more than 150 μV was rejected. Finally, the signal was filtered with a band-pass of 0.01–45 Hz. To confirm that the meditators were in the meditative state during the second session, EEG spectral analysis was carried out for the entire 9.24 min for resting state and for the entire 9.24 min for meditative state. A fast fourier transform (FFT) was used to calculate spectral power density (μV^2^).

#### EEG Feature Extraction

The EEG microstates were derived from the resting EEG data using sLORETA software. To compute microstates the local maxima of the global field power (GFP; Lehmann and Skrandies, 1980) were identified. Since scalp topography remains stable around peaks of GFP as a result of temporal smoothing in sLORETA, they are representative of the transient microstates (Pascual-Marqui et al., 1995; Koenig et al., 2002). The algorithm implemented for estimating microstates is based on a modified version of the classical k-means clustering method in which cluster orientations are estimated. An optimal number of 13 clusters for this method was determined by means of a cross-validation criterion (Pascual-Marqui et al., 1995). The entire EEG data at each time point was decomposed into a temporal sequence of one of these 13 EEG microstates. We used this EEG microstate decomposition to guide the analysis of the rsfMRI data at the single subject level.

#### rsfMRI Preprocessing

The rsfMRI analysis was performed using FSL software (FMRIB’s Software Library^1^), in particular with the fMRI Expert Analysis Tool (FEAT) and multivariate exploratory linear decomposition into independent components (MELODIC) modules. The first five functional images (EPI volumes) were discarded from each of the subjects’ rsfMRI data to allow for signal equilibration, giving a total of 180 volumes used in analysis. We conducted motion correction using MCFLIRT (Jenkinson et al., 2002), and non-brain tissue (Scalp, CSF, etc.) removal using the Brain Extraction Toolbox (Smith, 2002). The average head motion of the subjects was also not found to significantly differ between the groups. Spatial smoothing was performed using a Gaussian kernel of 5 mm full width at half maximum (FWHM), followed by a mean based intensity normalization of all volumes by the same factor, and then a high temporal band-pass filtering with sigma 180 s. These temporal filtering parameters were based on previously validated methodology employed by Beckmann and Smith (Smith et al., 2004; Beckmann and Smith, 2005; Beckmann et al., 2005). We co-registered the EPI volumes with the individual subject’s high-resolution anatomical volume in MNI152 standard space template and re-sampled the filtered data into standard space, keeping the re-sampled resolution at the fMRI resolution (3 mm) using FNIRT, the FMRIB non-linear image registration tool (Klein et al., 2009). Two participants from each group were excluded due to uncorrectable cardio-ballistic and motion artifacts during data preprocessing.

#### Identification of DMN-Microstate

The spectral Z-stats maps of the EEG microstates were created by convolving all the 13 microstates with the gamma hemodynamic response function using customized three column format in FEAT Version 5.98, part of FSL (FMRIB’s Software Library^2^). The GLM was modeled as an event related design, where EEG microstates were considered as explanatory variables (regressors) for rsfMRI analysis. This procedure, enabled us to combine EEG and fMRI time series despite their very different sampling rates. To do so, the onset time and the duration of each EEG microstate were provided as inputs to the GLM and convolved with a gamma hemodynamic response function (Musso et al., 2010). This rsfMRI, thus informed by the EEG microstates, was analyzed at the individual level to derive subject-wise Z-stats maps for each microstate. We examined the spatial correlation (R) between the spectral Z-stats map and the DMN component estimated by ICA of blood-oxygen-level dependent (BOLD) activity (see below). Using the FSL utility “fslcc”, the Z-stats maps which correlated with BOLD ICA map of the DMN with a correlation of at least 0.3 were selected for further analysis (Kiviniemi et al., 2011). When more than one microstate Z-map correlated with the DMN the one with highest correlation was selected. This subject-wise spectral Z-stats map of the DMN microstate was included in further group analysis. Group level analysis was done using the higher level FEAT tool, where three group mean effects (controls at rest, meditators at rest and meditation state) were extracted by specifying the contrast (1 0 0; 0 1 0; 0 0 1) respectively.

#### Independent Components (ICs)

Functional connectivity was ascertained using ICA which decomposes the rsfMRI data into statistically independent spatial maps (and associated time series). This is a data driven approach by which we can extract the functional networks in a voxel-wise manner based on their spatial and temporal signal fluctuations. ICA of rsfMRI was carried out with the probabilistic independent components analysis (PICA) using FSL’s MELODIC method. This single-subject ICA was carried out with FEAT MELODIC data exploration, in which the rsfMRI data was extracted into the independent components (ICs) during the preprocessing step. All extracted IC were visually inspected. Non-motion related noise sources such as eye related artifacts, field in homogeneity, high frequency noise, slice dropout and gradient instability were removed on the basis of existing literature (Smith et al., 2004; Beckmann and Smith, 2005; Beckmann et al., 2005). ICs that showed clearly identifiable patterns in both spatial and frequency domains, and activations in frequency plots below 0.1 Hz were removed to reduce respiration and heart rate related artifacts. The selected noise components were removed using the command “fsl_regfilt” defined in FSL MELODIC. Multivariate group PICA was then carried out using FSL MELODIC (Beckmann and Smith, 2005; Beckmann et al., 2005), to derive maximally spatially ICs across all datasets (18 control and 18 meditator × 2 sessions), which were temporally concatenated. The data set was decomposed into 15 sets of independent vectors which describe signal variation across the temporal (timecourses) and spatial (maps) domain by optimizing for non-Gaussian spatial source distributions using the Fast ICA algorithm. The resulting maps were threshold using an alternative hypothesis test with a significance level of *p* > 0.5 (i.e., an equal weight is placed on false positives and false negatives) by fitting a Gaussian/Gamma mixture model to the histogram of intensity values (Smith et al., 2004; Beckmann and Smith, 2005). Finally, the components representing the DMN were visually identified from the set of group IC maps by comparing their constituent cortical sources with the regions of the DMN reported in the literature (Beckmann and Smith, 2005; Beckmann et al., 2005; Damoiseaux et al., 2006).

#### DMN-Microstate Statistical Analysis

The mean duration and number of occurrences per second of the DMN microstates were calculated for each subject. The duration of the DMN-microstate was defined as a continuous time epoch (milliseconds) during which all EEG GFP maps were assigned to the the DMN-microstate class by the k-means clustering algorithm. The frequency of occurrence of the DMN-microstate is defined the number of such occurrences per second. Group differences were calculated for both the DMN-microstate duration and frequency of occurrence for all subjects. Independent samples *t*-tests were carried out to compare the difference between meditators and controls at rest, and a paired sample *t*-test was carried out between the meditator at rest and during meditation. At a 95% confidence interval (CI), a *p*-value of < 0.05 was considered significant. Furthermore, the number of years of meditation practice were correlated with the duration and occurrence of DMN microstates with a pearson correlation. Multiple comparisons were compensated for by FDR correction.

#### DMN Dual Regression Analysis

Groupwise comparison of rsfMRI ICs was performed using dual regression, which allows for voxel-wise comparisons^3^. The set of spatial maps from the group-mean analysis (GroupICs) was used to generate subject-specific-spatial maps and associated time series (Beckmann et al., 2009; Filippini et al., 2009). DMN component maps from all subjects were arranged into a single 4D file and voxel-wise differences were estimated. Statistical differences were assessed using randomized, on-parametric permutation, incorporating the threshold-free cluster enhancement (TFCE) technique (Smith et al., 2009). Separate null distributions of *t*-values were derived for the contrasts reflecting the inter-group effects by performing 5000 random permutations and testing the difference between groups or against zero for each iteration (Nichols and Holmes, 2002). To estimate group mean effects, HC > meditator, meditator > HCs, meditator > meditation and mediation > meditator contrasts were used and the resultant statistical maps were threshold at *p* < 0.05 (family-wise error (FWE) corrected). Following this, inter-group effects were threshold by at *p* < 0.05 (false discovery rate (FDR) corrected; Filippini et al., 2009). When the voxel wise differences between the groups were higher, the term “increased connectivity” was used and vice versa.

## Results

Despite being in an MRI scanner with gradient acoustic noise, with a fixed head and body position, all meditators reported that they were able to stay awake in relaxed state and also enter into a meditative state as instructed. Visual inspection of the EEG for sleep spindles and K-complexes confirmed that none of the subjects fell asleep during rsfMRI data acquisition. In all the results presented below, three conditions are compared, controls at rest, meditators at rest, and the same meditators during meditation.

### Group Level EEG Frequency Differences During Meditation

**Figure 1:**
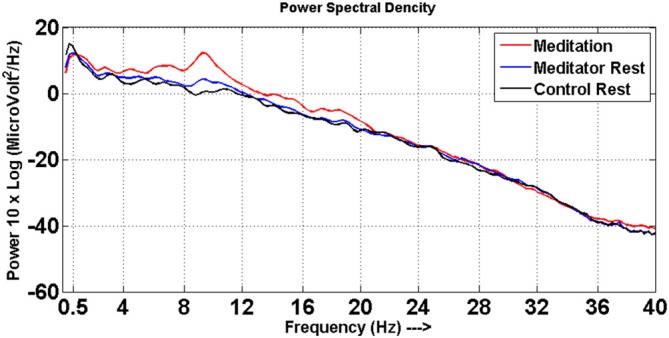
**Power spectra.** Power spectral density distribution for meditators at meditative state (red line) and at rest (blue line), control at rest (black line).

Significant changes were noted in spectral power of EEG frequency bands between the meditation and rest states in meditators. Meditators had increased power particularly in the alpha, theta and beta frequencies in the meditation state compared to rest state, which is depicted in the Figure 1. This also ascertained that the meditators did not go into meditative state during resting state and performed meditation in meditative state as instructed.

### Spatial Analysis of DMN-Microstate and DMN During Meditation

**Figure 2:**
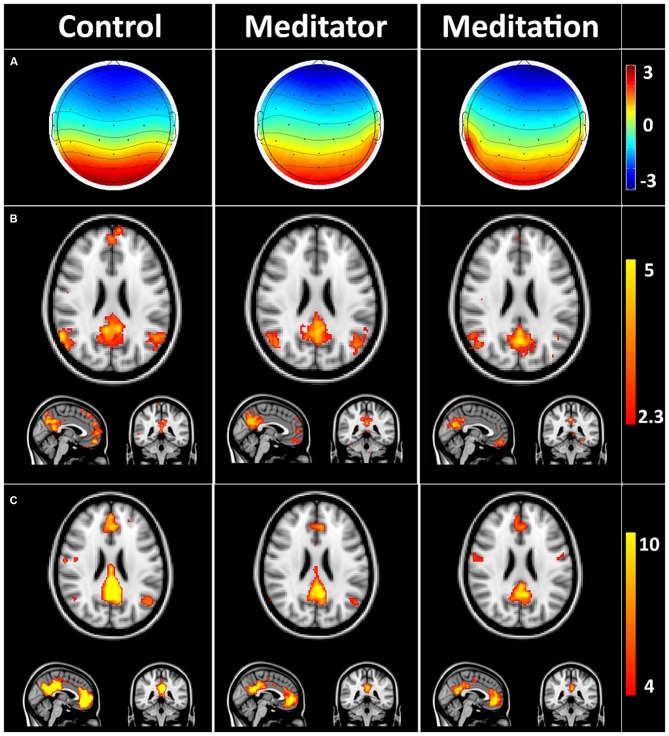
**Default mode network (DMN) microstates during rest and meditation. (A)** Group averaged electroencephalography (EEG) microstate in controls at rest (control), meditator at rest state (meditator) and meditator during meditation (meditation). **(B)** Group averaged Z-stats maps corresponding to the DMN microstates. **(C)** Corresponding DMN components derived from resting state functional magnetic resonance imaging (rsfMRI) using independent components analysis (ICA). The striking spatial similarity between the EEG-derived DMN Z-stats map **(B)** and rsfMRI-derived DMN **(C)** is evident. Meditators showed evidence of decreased connectivity in precuneus/posterior cingulate cortex (PCC) and medial prefrontal cortex (mPFC) at rest, which decreased further during meditation **(C)**.

Figure 2 depicts the average DMN-microstate, the corresponding Z-stats map and rsfMRI-derived DMN ICA component in HCs, meditators at rest (meditator) and during meditation.

**Figure 3:**
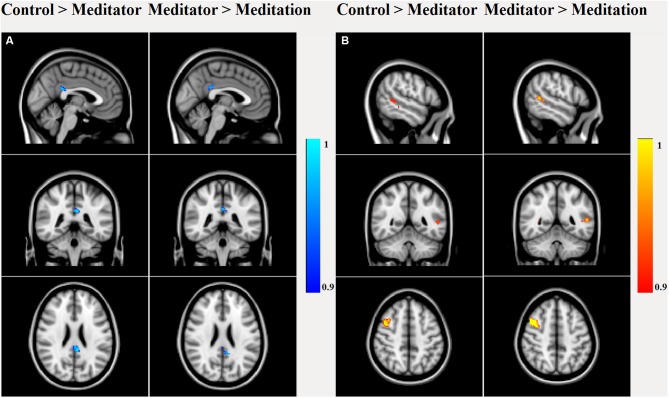
**Dual regression analysis of group main and between-group effects in the rsfMRI DMN component.** Between-group effects showed significant (*p* < 0.05 false discovery rate (FDR) corrected) decreased PCC connectivity in meditators compared to controls, which further decreased during meditation. **(A)** Meditators had higher connectivity in right middle frontal gyrus (MFG) and left middle temporal gyrus (MTG) than controls, which again increased during meditation **(B)**.

Dual regression analysis (Figure 3) of the rsfMRI DMN revealed that meditators were different from HCs (*p* < 0.05) already at rest, as they had decreased posterior cingulate (number of voxels: 223, T-max: 0.987, coordinates: −4, −40, 26) connectivity, increased right middle frontal gyrus (MFG; number of voxels: 263, T-max: 0.956, coordinates: 42, 8, 46) and left middle temporal gyrus (MTG; number of voxels: 53, T-max: 0.963, coordinates: −52, −48, 6) connectivity. This relatively lower cingulate connectivity was further significantly reduced relative to rest, when the meditators entered into the meditative state (number of voxels: 107, T-max: 0.998, coordinates: −2, −40, 26). Alongside this reduction, we also observed increased left MTG (number of voxels: 122, T-max: 0.992, coordinates: −58, −46, 8) and right MFG (number of voxels: 424, T-max: 0.998, coordinates: 40, 8, 46) connectivity during meditation.

### Temporal Analysis of the DMN-Microstate

**Figure 4:**
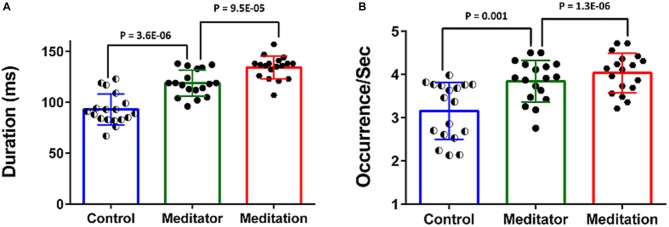
**Temporal dynamics of the DMN-microstate.** Bar charts plot the duration **(A)** and occurence/s **(B)** of the DMN-microstate in healthy controls (HCs), meditators at rest and during meditation. Both measures were significantly higher in meditators than in controls, and increased further during meditation.

The meditators at rest had increased duration and higher frequency of occurrence of the DMN-microstate compared to controls (Figure 4).The mean duration of the DMN-microstate was 93.05 ± 15.18 ms in HCs and 118.88 ± 12.78 ms in meditators at rest (Unpaired *t*-test, *t* = −3.55; df = 34; *p* = 3.6E-06); the mean frequency of its occurrence was 3.15 ± 0.66/s in controls and 3.84 ± 0.48/s in meditators (Unpaired *t*-test, *t* = −3.56; df = 34; *p* = 0.001). During meditation the duration and frequency of occurrence increased significantly further. Specifically, during meditation, the mean duration of DMN-microstate was 134.27 ± 11.21 ms (Paired *t*-test, *t* = −5.06; df = 17; *p* = 9.5E-05) and the mean frequency of occurrence was 4.03 ± 0.46/s (Paired *t*-test, *t* = −5.06; df = 17; *p* = 1.3E-06; Figure 4).

### Correlation of DMN-Microstate Dynamics With Years of Experience in Meditation

**Figure 5:**
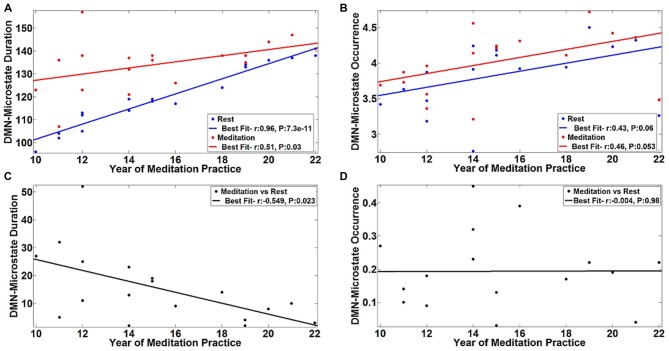
**Correlation of DMN-microstate dynamics with meditation experience.** The duration **(A)** and frequency of occurrence **(B)** of the DMN-microstate were positively correlated with years of experience. The blue and red lines in dicate the linear fits of these DMN-microstate properties measured during meditation and rest, respectively. Duration was less altered as a function of state in more experienced meditators. Corroborating this trait effect, the difference in DMN-microstate duration between meditation and rest was negatively correlated with years of experience **(C)**. No such trait effect was observed in the frequency of occurrence of the DMN-microstate **(D)**.

The observed differences in the temporal properties of the DMN-microstate suggested a combination of state and trait-based influences. To further explicate the influence of long-term meditation experience in engendering trait differences between meditators and controls, we correlated the duration and frequency of occurrence of the DMN-microstate in individual meditators with their meditation experience in years. The results are summarized in Figure 5. There was a very strong, significant positive correlation in the duration of DMN-microstate in meditators at rest (*r* = 0.96, *p* = 7.3E-11) and in the meditative state (*r* = 0.51, *p* = 0.03) with years of meditation practice (Figure 5A). Interestingly, in each meditator, the net increase in the duration of the DMN-microstate during meditation relative to rest actually correlated negatively with the years of meditation practice (Figure 5C, *r* = −0.549, *p* = 0.023). This implied that the more experienced meditators had a DMN-microstate duration akin to being in a meditative state even at rest, highlighting how the state effect of meditation on DMN dynamics gradually transitions into a trait effect that potentially permanently alters it over many years of practice. However, this effect could potentially be explained away by the age of the meditators rather than their years of experience, as participants with longer years of experience were older than those with lesser experience. To address this potential confound, we regressed out this effect by including age of the meditator as a covariate in the correlation analysis. Having accounted for the influence of age, we still found a significant negative correlation between the state-induced change in DMN-microstate duration and years of mediation experience (*r* = −0.549, *p* = 0.03). This confirmed that there was indeed a trait-level modulation of the DMN’s dynamics produced by meditation experience, that could not be explained away by aging.

Further, we found that this pattern of effects were specific to the duration of the DMN-microstate. Specifically, the effect of meditation experience on the frequency of occurrence of the DMN-microstate was not statistically significant, either at rest (*r* = 0.43, *p* = 0.06) or in the meditative state (*r* = 0.46, *p* = 0.053), though there was a trend towards increasing DMN-microstate occurrence with increasing years of meditation practice (Figure 5B). Further, the meditation state induced increase in the frequency of occurrence did not correlate with years of practice (*r* = −0.004, *p* = 0.98). As the frequency of occurrence of the DMN-microstate was unaffected by the years of experience, it was distinct from duration and is probably indicative of the state effect of meditation, as seen in Figure 4B.

## Discussion

We have investigated dynamic alterations of the DMN using simultaneous EEG-fMRI, with the aim of characterizing the spatial and temporal changes caused by meditation induced alterations of consciousness. Using spatial ICA analysis of our fMRI data, we found that the posterior cingulate hub of the DMN was less strongly connected in meditators, which became further reduced during meditation. In contrast, the right middle frontal and left middle temporal gyri were more active in meditators and during meditation. By linking the fMRI with EEG, we were able to identify the DMN microstate in all participants, by matching its spatial configuration to their respective DMN ICA components. The temporal properties of this DMN-microstate highlighted its significantly higher duration and frequency of occurrence, in meditators at rest and during meditation. Further, the relationship between these temporal properties and years of meditation experience enabled us to explore state and trait influences on DMN dynamics. This analysis suggested that in less experienced meditators, entering into a meditative state was associated with significant increases both in the duration and frequency of the DMN-microstate. As experience with meditation increased, there was a progressively higher duration of the DMN-microstate at rest and entering a state of meditation only brought about an increase in its frequency of occurrence. Taken together, these findings elucidate salient spatiotemporal mechanisms of meditation, and particularly highlight its role in altering the temporal dynamics of brain activity over short and long time scales.

Within the large body of fMRI literature on resting state networks, the DMN has been a particular focus of attention (Raichle et al., 2001; Buckner et al., 2008) as a key brain network that underlies awareness of the self within the environment. Our finding of decreased PCC connectivity and increased temporal and frontal connectivity within the DMN during meditation has been reported previously in seed to voxel connectivity studies (Farb et al., 2007; Brewer et al., 2011). Alongside, the significance of the temporo-frontal regions during meditation has been consistently highlighted in previous task based fMRI studies (Brefczynski-Lewis et al., 2007; Lagopoulos et al., 2009). Indeed, our findings mirror those from recent studies on fMRI of meditation (Baijal and Srinivasan, 2010; Cahn et al., 2010; Hasenkamp and Barsalou, 2012; Hasenkamp et al., 2012). In particular, Hasenkamp and Barsalou (2012) studied the effects of meditation experience on DMN functional connectivity, and demonstrated increased connectivity in frontal areas during meditation in meditators with more experience, which they interpreted as evidence of greater attentional control gained with such experience. In our meditator cohort, these attentional control networks could also be similarly more active in the expert meditators. This increased attentional control, along with decreased PCC connectivity, is hypothesized to undergo neuroplastic changes that enable them to gain better control of mind-wandering. Based on evidence of task positive networks being recruited at rest, it has been argued that continuous and regular meditation practice transforms the rest state of experienced meditators (Brewer et al., 2011). However in the current study the right frontal and left temporal areas described are part of the DMN and do not represent task positive networks. However, it is possible that this neuroplasticity is mediated by task positive networks, and future research could elucidate how meditation modulates the relationship between these different brain networks.

Within this research context, our finding have generated new evidence delineating temporal changes in DMN activity that accompanies these spatial changes. Our finding of increasing frequency of occurrence of the DMN-microstate during meditiation implies that there is a decreased occurrence of other microstates, thus resulting in an overall decrease in variability in the temporal dynamics of brain activity. According to large scale dynamical models of consciousness (Deco et al., 2013), resting brain can be divided into three states, namely the spontaneous state, multistable attractor states and unstable spontaneous state, differing in their coupling strengths. The state with least coupling strength is the spontaneous state and that with highest coupling strength is the unstable spontaneous state often associated with a task. Human consciousness is postulated to be positioned at the verge of instability defined to lie between the multistable attractor state and the unstable spontaneous state. Within this framework, decreased variability within such a dynamical system is compatible with our finding of increased frequency of the DMN microstate during meditation. It is worth noting that the focus of the analysis here has been on the DMN-microstate, as that was the only microstate that was consistently present in all subjects. This is similar to findings reported by Musso et al. (2010), whose group analysis only revealed a fronto-occipital network that overlapped with visual networks and the DMN. This is despite the fact that all the subjects in their study had microstates which correlated with several other resting state networks. With the continual advancement in methodological tools for simultaneous EEG-fMRI analysis, we might be able parse spatiotemporal brain dynamics with the requisite detail and consistency for mapping all the resting state networks.

Our finding of experienced meditators having a higher duration of DMN microstate that did not change much in the meditative state is consistent with the philosophical perspective (Lutz et al., 2008), behavioral (Carter et al., 2005; Srinivasan and Baijal, 2007) and brain structural changes (Lazar et al., 2005) related to the trait effects of meditative practice. Because of the synergistic interaction between state and trait effects it has been largely difficult to design studies to separate these effects (Cahn and Polich, 2006) and hence relatively few studies that have exclusively looked at the state effect of meditation. In future research, the study of neurophenomenology of meditation, which correlates moment-to-moment first person reports of internal experience to guide neuroimaging analysis might be able to separate state effect from trait effects of meditation (Jack and Shallice, 2001; Jack and Roepstorff, 2002; Lutz et al., 2002).

## Conclusion

Our investigation of the spatial configuration of the DMN in meditators and during meditation revealed a decrease in posterior cingulate connectivity, and a complementary increase in middle frontal and temporal connectivity. Using temporal microstate analysis applied to simultaneous EEG-fMRI data, we found that the duration and frequency of occurrence of the DMN-microstates are useful metrics of meditation-induced altered consciousness, and increase systematically across controls, meditators during rest and during meditation. We report complementary roles of these metrics, where duration primarily reflected the trait effect and occurrence represented the state effect of meditation. Our results shed new light on the neurobiology of meditation, and its effect on the spatiotemporal properties of the DMN in particular.

## Author Contributions

RP and RDB have contributed equally for this article. Contributions to the conception or design of the work by RP, RDB, SLR. Data acquisition done by RP and NU and analysis was carried out by RP, RDB, NU, SM. RDB, SC interpreted the results of the study. Manuscript drafted by RP followed by RDB, SC, NU revised the manuscript critically for important intellectual content. Final approval and agreement to be accountable for all aspects of the work in ensuring that questions related to the accuracy or integrity of any part of the work are appropriately investigated and resolved by all authors.

## Funding

We acknowledge the support of the Department of Science and Technology, Govt. of India, India for providing the 3T MRI scanner exclusively for research in the field of neurosciences.

## Conflict of Interest Statement

The authors declare that the research was conducted in the absence of any commercial or financial relationships that could be construed as a potential conflict of interest.

## Footnotes

^1^www.fmrib.ox.ac.uk/fsl

^2^http://fsl.fmrib.ox.ac.uk/fsl/fslwiki/

^3^http://fsl.fmrib.ox.ac.uk/fsl/fslwiki/DualRegression
